# Tree type-specific endophytic bacterial assembly and function in senescing leaves and needles in temperate forests of Central Europe

**DOI:** 10.1186/s12870-026-08931-x

**Published:** 2026-05-21

**Authors:** Li Ji, Benjawan Tanunchai, Simon Andreas Schroeter, Sara Fareed Mohamed Wahdan, Shakhawat Hossen, Matthias Noll, Witoon Purahong

**Affiliations:** 1 School of Forestry, Central South University of Forestry and Technology, Changsha, 410004 P.R. China; 2 Department of Ecology of Agroecosystems, UFZ-Helmholtz Centre for Environmental Research, Theodor-Lieser-Str. 4, Halle (Saale), 06120 Germany; 3 Department of Applied Natural Science and Health, Institute of Bioanalysis, University of Coburg, Coburg, Germany; 4 Biogeochemical Processes Department, Max Planck Institute for Biogeochemistry, Hans-Knöll-Str. 10, Jena, 07745 Germany; 5 Department of Botany and Microbiology, Faculty of Science, Suez Canal University, Ismailia, 41522 Egypt

**Keywords:** Leaf-associated bacteria, Tree type, Assembly process, Endophytes, Co-occurrence network, Central European forests

## Abstract

**Background:**

Leaves and needles are pivotal plant tissues in regulating carbon input, nutrient uptake, and biogeochemical cycling within ecosystems. However, the ecological strategy of broadleaved and coniferous tree species affecting the endophytic colonization of leaf/needle for enabling a nutrient release of subsequent litter is not fully known. The bacterial community assembly, functions, and interactions (summarized as attributes) inhabiting senescing leaves and needles in eleven common tree species of Central European forests were investigated using next generation sequencing.

**Results:**

Endophytic bacterial attributes varied significantly among tree species and between tree types (broadleaved and coniferous trees). Deterministic processes (nitrogen (N) related factors) governed the endophytic bacterial community assemblages in broadleaved tree species, which were dominated by ureolytic bacteria that potentially caused the increase in inorganic N. In turn, stochasticity (homogenizing dispersal and dispersal limitation) predominantly controlled the community assembly of bacteria inhabiting in needles. Compared with broadleaved, coniferous trees exhibited both more diverse bacterial taxa enlarging the capacity to cope with changing environmental factors.

**Conclusions:**

Our results reveal the endophytic colonization patterns that initiate litter decomposition during leaf and needle maturation and demonstrate variations between tree types.

**Supplementary Information:**

The online version contains supplementary material available at 10.1186/s12870-026-08931-x.

## Background

Terrestrial plants are among the most significant contributors to biomass globally, accounting for approximately 450 Gt carbon (C) [1]. Large fractions of terrestrial plant biomass (about 150 Gt C) are stored in the roots and leaves of woody plants [2]. Apart from C, leaves also contain nitrogen (N), potassium (K), phosphorus (P), and other trace elements acquired from soils. The amounts of these nutrients in leaves are much higher than in woody stems [3]. Thus, leaves can be considered a critical nutrient pool in forest ecosystems due to the limited lifespan of leaves, which has significant impacts on forest nutrient cycles and primary productivity [4]. Plant leaves in terrestrial ecosystems provide diverse habitats for microbiota, both internally (as endophytes) and externally (as epiphytes) [5]. The total number of both endo and epiphytic microbes on the earth exceeds 10^26^ cells, and a typical leaf may contain up to 10^6^–10^7^ bacteria per square centimeter of surface [6, 7]. To differentiate endophytic and epiphytic leaf washing procedures and controls were carried out [8]. Due to their aerial nature, leaves are directly exposed to periodic fluctuations in temperature, humidity, and ultraviolet radiation combined with rain washing, wind-mediated movement, insect and animal damage, and environmental pollution and are therefore considered transient habitats with harsh and hostile conditions for microorganisms [9]. Thus, the diversity and abundance of endophytic microbial communities are inferior to those of root-associated soil microbes [10].

Endophytic microbiota can mediate the fitness of their host plants [11], by increasing the resistance to abiotic stress, reducing the negative effect of herbivory on the plants, and promoting nutrient cycling [12]. Previous studies have demonstrated that leaf-inhabiting bacteria are widely mediated in species and function [13, 14], including dinitrogen (N_2_) fixation [15], plant growth promotion [16, 17], plant hormone production, interactions with plant pathogens [16], and environmental pollutant degradation [18]. There are indications that leaf litter decay begins on mature leaves still attached to plants [4]. It is unclear whether such variations occur in endophytic bacteria, but it has been shown that the microbiome associated with mature leaves plays a significant role in leaf decomposition, influencing microbial succession during later stages of decay [19].

Understanding the relative importance of stochastic (e.g., dispersal limitation, ecological drift) versus deterministic (e.g., environmental filtering, species interactions) processes is fundamental to explaining specific bacterial community structures, compositions, and potential functions [20]. The endophytic bacterial community, without exception, is regulated by deterministic and stochastic processes, but there is considerable controversy regarding the trade-off between them [21]. The deterministic process based on the niche theory is primarily governed by spatial accessibility for microbes, interspecies interaction, and environmental filtration, while dispersal limitations, ecological drift, and diversification affect the stochastic process of microbial communities [22]. Moreover, much uncertainty remains regarding the relative importance of their assembly processes. Maignien et al. [23] suggested that the *Arabidopsis*-associated leaf bacterial community initially reflects the airborne community (stochastic event), and as the plant matures, the interaction between host and bacterial members forms a niche that is conducive to the survival of specific taxa (deterministic event). The highly connected microbial taxa, which dominate community structure and functions, were identified as critical for the fitness and function of the host [24, 25]. The specific ecological effects of these interactions, as well as the potential function of microorganisms in their habitats, remain to be uncovered [24].

Mounting evidence has shown that leaf physicochemical traits, such as nutrient composition (C, P, N, and soluble sugar content) and water content affect the colonization of endophytic microbes, however, living leaves are not a viable nutrient source for epiphytes due to the cuticle preventing access to nutrients [26, 27]. While leaves before apoptosis provide a very low nutrient availability for external microbial community diversities, Vorholt [28] suggested that the chemical properties of leaves had a significant effect on endophytic growth and survival. Yadav et al. [26] revealed that the leaf water content and P content were primary drivers of endophytic abundance in trees and shrubs in the Mediterranean area. In addition to leaf structure, chemical composition, and exudates [29, 30], plant identity and genotype, spatial location, and leaf growth stage can also affect endophytic as well as epiphytic microbial community composition [31–33]. Laforest-Lapointe et al. [34] and Lajoie et al. [35] demonstrated that the host identity was the highest impact factor affecting endophytic bacterial community composition in temperate and neotropical trees. One key gap in our knowledge is how all these factors interact with each other and how they mediate endophytic bacterial community assembly. Shape of leaves is important for leaf litter decomposition as it is linked to leaf N contents and resource-use strategies [36]. Nevertheless, N content may not be the only factor explaining increased leaf litter decomposition rates with accompanying broadleaved trees, which is governed by other environmental factors and litter traits (e.g., lignin: N ratio, saprotrophic fungi, and initial magnesium concentration, etc.) [37–39]. Alternatively, coniferous trees improve soils relatively rich in organic nutrients due to the low-quality litter and slow mineralization rate of soil nutrients [40]. Despite advancements in characterizing endophytic bacterial communities in numerous tree species, a crucial knowledge gap persists concerning the comparative and mechanistic roles of tree type (broadleaved vs. coniferous) and specific leaf physicochemical properties in determining the diversity, community structure, and underlying assembly processes of these microbes across diverse hosts within Central European forest ecosystems.

Our recent study revealed that a less connected network of cross-kingdom was observed at the initial stage of litter decomposition than those at 200 d and 400 d, which is more sensitive to external perturbations [41]. These sensitive underscores the importance of exploring the bacterial community attributes within senescent leaves or needles, which is crucial for understanding subsequent decomposition processes. Following the theory of priority effects, during early colonization, an initial colonizer can seize and prevent later-arriving species from occupying niches by outcompeting them for resources [42]. Nonetheless, previously published studies are limited in examining the contrasting patterns of community assembly processes and functional profiles of endophytic bacteria in broadleaved and coniferous trees. The aims of this study were i) to explore the bacterial attributes (diversity, community composition and assembly) of mature plant leaves and needles, and their relationship with tree types (broadleaved and coniferous trees) of respective tree species; (ii) to investigate any correlation between endophytic bacterial attributes and variations in leaf physicochemical properties; (iii) to link bacterial functions to tree types. Our main hypotheses are that tree types significantly influence the bacterial attributes of mature leaves and that these attributes are strongly correlated with leaf physicochemical properties.

## Materials and methods

### Study site and sampling

The study was conducted in the Hainich–Dün region of Thuringia, Germany (51°12′ N, 10°18′ E), encompassing an altitudinal range of 100 to 494 m above sea level. Mean annual precipitation in the area varies between 600 and 800 mm, with a mean annual temperature ranging from 6.0 to 7.5 °C. The pedological profile is dominated by Cambisol, a soil type formed on a limestone bedrock, with an average soil pH of 5.1. In October 2019, senescing leaves or needles of eleven temperate tree species (9 tree species ⋅ 5 independent tree replicates + 1 tree species (*Prunus avium*) ⋅ 3 independent tree replicates + 1 species (*Larix decidua*) ⋅ 4 independent tree replicates = 52 individual tree samples; Supplementary Table S1) were collected from a forest in Thuringia, Germany (Supplementary Fig. S1). The mature leaves and needles were at the last step of the senescence before them fell. The specimens were harvested from the lower branches of the mature tree crowns. These eleven temperate tree species include seven broadleaved tree species [*Acer pseudoplatanus* (AH), *Fagus sylvatica* (BU), *Quercus robur* (EI) *Fraxinus excelsior* (ES), *Carpinus betulus* (HBU), *P. avium* (KB), *Tilia cordata* (LI)] and four coniferous tree species [*Pseudotsuga menziesii* (DG), *Picea abies* (FI), *Pinus sylvestris* (KI), *L. decidua* (LA)]. Notably, two samples from *P. avium* (KB) and one sample from *L. decidua* (LA) were lost due to failure (the generation of an insufficient number of high-quality sequences) or outlier of bacterial analysis. The trees were sampled from sites with similar environmental conditions (e.g., same bed-rock, weather conditions, biotic and abiotic stressors), forest management practice, and soil texture. For each individual tree, mature leaves (1-year-old) and needles (3-year-old) were sampled separately using clean gloves and placed into sterilized plastic bags. A minimum of 200 g of leaf or needle mass was collected per tree. The samples were further divided into two subsets: one for physicochemical analyses was stored at 4 °C, while the other was transported on ice to the laboratory within 3 h and preserved at − 80 °C pending further processing.

### Measurements of physicochemical properties

The measurements of leaf properties followed the description in our previous publications [40, 42]. Briefly, wet leaf and needle samples were incubated with 30 mL of Milli-Q water for 1 h to extract water-soluble components from their surfaces. All quantification results are reported relative to the dry weight of the leaves or needles. The pH of the leachates was measured using pH indicator paper with a resolution of 0.2 pH units [43]. Organic nitrogen (N_org_) was quantified using a sum parameter analyzer employing high-temperature combustion and chemiluminescence detection (Mitsubishi TN-100; Düsseldorf, Germany). Ammonium (N_NH4+_), nitrite (N_NO2−_), and nitrate-plus-nitrite (N_NO3− + NO2−_) were determined via a flow injection analyzer (Quikchem QC85S5; Lachat Instruments, Hach Company, Loveland, CO, USA), utilizing appropriate manifolds for each analyte. Detailed protocols for the measurement of leaf and needle variables are provided in the Supplementary Information.

### DNA extraction and illumina sequencing

Following sample collection, mature leaves and needles were meticulously separated from branches to prevent cross-contamination in the laboratory. Up to 10 leaves and needles from five branches per tree individual were randomly selected for DNA extraction preparation. To eliminate dust particles and loosely adherent surface microbiota, leaf and needle samples were washed three times by vortexing in sterile Tween solution (0.1% v/v) at maximum speed for 5 min. Subsequent rinsing with deionized water, performed three to five times or until no visible bubbles were observed, removed residual Tween solution. Each composite sample was homogenized using liquid N_2_ and sterilized tools. Approximately 120 mg of the homogenized leaves and needles underwent DNA extraction with the DNeasy PowerSoil Kit (Qiagen, Hilden, Germany) and a Precellys 24 tissue homogenizer (Bertin Instruments, Montigny-le-Bretonneux, France), following the manufacturer’s protocols. Genomic DNA presence and concentration were verified via NanoDrop ND-1000 spectrophotometry (Thermo Fisher Scientific, Dreieich, Germany), and the extracts were cryopreserved at − 20 °C.

Leaf- and needle-endophytic bacteria were profiled using 16 S rRNA gene-based amplicon sequencing on the Illumina MiSeq platform [44]. Bacterial amplicon libraries were generated by amplifying the hypervariable V4 region of the 16 S rRNA gene with the universal bacterial/archaeal primer pair 515 F (5′-GTGCCAGCMGCCGCGGTAA-3′) and 806R (5′-GGACTACHVGGGTWTCTAAT-3′), incorporating Illumina adapter sequences [45]. Detailed PCR composition and cycling conditions are described in Tanunchai et al. [41]. Amplifications were conducted in 20 µL reaction volumes using 5× HOT FIRE Pol Blend Master Mix (Solis BioDyne, Tartu, Estonia). Amplified products were visualized by gel electrophoresis and purified with the Agencourt AMPure XP kit (Beckman Coulter, Krefeld, Germany). Illumina Nextera XT Indices were ligated to both ends of the bacterial amplicons. Products from three technical replicates were pooled in equimolar concentrations. A pooled negative control from all PCR runs was included for sequencing as a quality control. Paired-end sequencing (2 × 300 bases) was performed on the pooled PCR products using a MiSeq Reagent Kit v3 on an Illumina MiSeq system (Illumina Inc., San Diego, CA, USA) at the Department of Soil Ecology, Helmholtz Centre for Environmental Research, Germany. A mock community was incorporated to validate sequencing accuracy, and bioinformatic filtering was applied to exclude reads derived from mitochondria and chloroplasts.

### Bioinformatics

Forward and reverse 16 S rRNA gene primer sequences were trimmed from demultiplexed raw reads using cutadapt [46]. Paired-end sequences were then subjected to quality trimming, chimera filtering, and merging using the DADA2 package [47] within the dadasnake pipeline [44]. High-quality assembled reads were retained for further analysis based on the following criteria: minimum lengths of 200 bp and 150 bp for forward and reverse sequences, respectively; quality scores ≥ 15 with a maximum expected error of 2 bp for both; and absence of ambiguous nucleotides. Merging permitted up to 2 mismatches and required a minimum overlap of 20 nucleotides for bacterial sequences. Following chimera removal, high-quality reads were clustered into 2,012 bacterial amplicon sequence variants (ASVs). Of these, 79 ASVs (4.06% of total) remained unclassified, comprising 1,171 reads (0.56% of total). The remaining 1,867 ASVs were classified to at least the phylum level. The ASV-based DADA2 approach offers superior resolution, along with comparable or enhanced sensitivity and specificity relative to conventional operational taxonomic unit (OTU) clustering methods, such as those employing 97% sequence similarity [48]. Taxonomic classification of bacterial ASVs was performed using the SILVA SSU database v. 138 [48], applying the Bayesian classifier implemented in the mothur classify.seqs command with a cutoff of 60. The ASV methodology facilitates inference of biological sequences within samples, as detailed previously [49]. Singleton ASVs (those comprising a single read across all samples), which may represent artifacts, were excluded. Negative PCR controls were sequenced alongside samples. Sequences detected in the pooled negative PCR control were removed from the dataset to mitigate technical errors or contamination. Rare ASVs (singletons or sequences found in only one sample) were then removed. The dataset was rarefied based on the vegan package in R with a minimum sequencing depth of 4,054 sequences per sample to minimize sequencing bias. Finally, 1,946 bacterial ASVs were obtained. Rarefaction curves for all samples demonstrated convergence, indicating sufficient sequencing depth (Supplementary Fig. S2). To infer functional potential, the ecological function of each ASV was determined using FAPROTAX [50]. the metabolic functional profiles of the endophytic bacterial communities within the nine temperate tree species were predicted using TAX4FUN2 implemented in R (v4.0.5) [51]. The complete bacterial taxonomic assignments are available in the Supplementary excel file.

### Statistical analysis

The rarefaction analysis was conducted in R using the vegan package (R Core Team, 2023). Specifically, the ‘*rarecurve*’ function was employed to generate individual rarefaction curves for each sample. Data normality and homogeneity of variance were assessed prior to analysis. Alpha (α)-diversity metrics were computed using PAST software. Differences in α-diversity among tree species were evaluated via Tukey’s honestly significant difference (HSD) test, while comparisons between broadleaf and coniferous trees employed an independent *T*-test. Beta (β)-diversity of endophytic bacterial communities was visualized through non-metric multidimensional scaling (NMDS) ordinations derived from Bray-Curtis dissimilarity matrices, implemented with the ‘vegan’ package in R (version 4.0.5). Permutational multivariate analysis of variance (PERMANOVA) was performed using the ‘adonis’ function within the ‘vegan’ package to examine variations in community composition across tree types and species. The effects of leaf physicochemical properties on endophytic bacterial communities were quantified via goodness-of-fit statistics based on abundance data and Bray-Curtis distances, also utilizing the ‘vegan’ package in R.

Taxonomic and metabolic networks for distinct tree types were constructed using the Molecular Ecological Network Analyses Pipeline (MENAP; http://ieg4.rccc.ou.edu/mena/), grounded in random matrix theory [52, 53]. For each empirical network, 100 randomized counterparts were generated, matched for network size and average number of links. Discrepancies between the observed network and these random networks were evaluated using *Z*-tests. Network topologies were rendered with Gephi (version 0.9.2). Keystone taxa were subsequently identified by calculating intra-module connectivity (*Zi*) and inter-module connectivity (*Pi*) for each node, applying established classification thresholds: (i) peripheral nodes (*Zi* < 2.5, *Pi* < 0.62), predominantly linked within their own modules; (ii) connectors (*Zi* < 2.5, *Pi* > 0.62), exhibiting strong inter-module linkages; (iii) module hubs (*Zi* > 2.5, *Pi* < 0.62), featuring extensive intra-module connections; and (iv) network hubs (*Zi* > 2.5, *Pi* > 0.62), combining characteristics of module hubs and connectors.

Sloan’s neutral community model (NCM) was conducted to determine the relative contribution of neutral processes to the community assembly by predicting the relationship between ASV occurring frequency and their abundance in a set of local communities [54]. The values of m and *R*^*2*^ indicated the migration rate and the overall fit to the NCM on the basis of nonlinear least-squares fitting, respectively. The 95% confidence intervals around all fitting statistics were calculated by bootstrapping with 1000 bootstrap replicates in R with the code provided by the description from Burns et al. [55]. The habitat niche breadth index was estimated to reveal the patterns of bacterial taxa sorting and dispersal limitation based on habitats and regions. The community-level of niche breadth was calculated as the average of values from all taxa occurring in one community [56]. Then, we further identified the ASVs as generalists or specialists based on their occurrence and by using permutation algorithms as implemented in the ‘EcolUtils’ package in R [57].

To elucidate the relative contributions of deterministic and stochastic processes to community assembly, we employed the phylogenetic normalized stochasticity ratio (pNST) and beta nearest taxon indices (βNTI), as described by the null model theory of Stegen et al. [58] and Ning et al. [20]. Complementing βNTI, the Raup-Crick (RC_bray_) null model, predicated on Bray-Curtis dissimilarities, was applied to quantify dispersal-driven stochastic processes underlying compositional turnover [59]. For pNST, a threshold of 50% demarcated predominantly deterministic assembly (pNST < 50%) from stochastic dominance (pNST > 50%). βNTI values below − 2 signified homogeneous selection, whereas values exceeding + 2 indicated variable selection. Dispersal limitation was inferred when |βNTI| < 2 and RC_bray_ > + 0.95, while homogenizing dispersal was identified by |βNTI| < 2 and RC_bray_ < − 0.95; the residual undominated fraction, encompassing diffusive stochasticity, was defined by |βNTI| < 2 and |RC_bray_| < 0.95 [59]. Both pNST and βNTI values, calculated using Bray-Curtis dissimilarity, were computed using the ‘iCAMP’ package in R, with the code provided by Ning et al. [20] (https://github.com/DaliangNing/iCAMP1).

## Results

### Endophytic bacterial community composition, diversity, and function: Significant differences among tree species and tree types

**Fig. 1:**
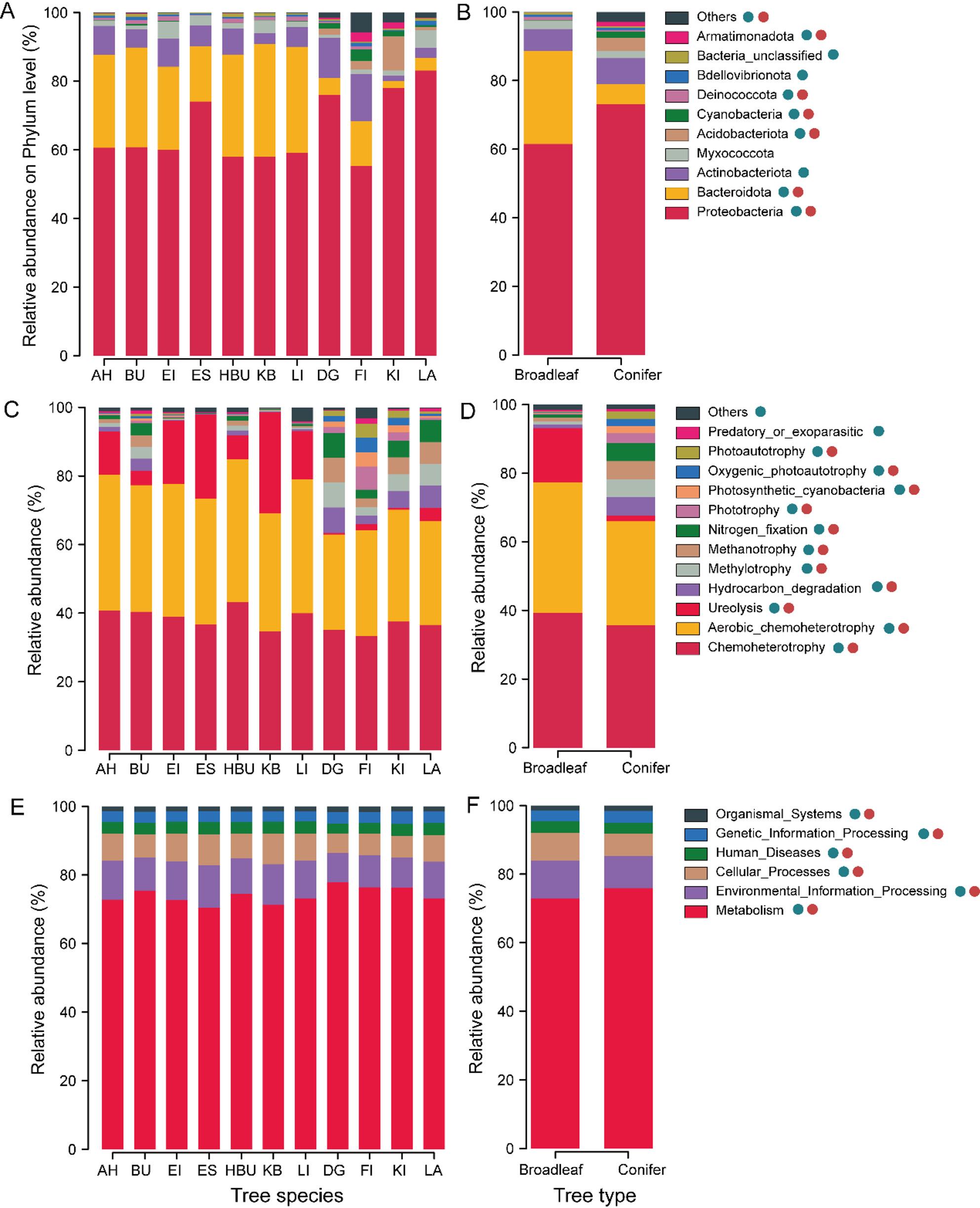
Taxonomic and functional composition of endophytic bacterial communities in eleven temperate tree species in Central European forests. **A**, **B** Phylum-level taxonomic composition of endophytic bacterial communities of tree species and tree types, respectively. Low abundance phyla with less than 0.5% of the total sequences across all samples are grouped into “Others.” **C**, **D** Ecological functions of endophytic bacterial communities of tree species and tree types, respectively. Low abundance function groups with less than 0.5% of the total sequences across all samples are grouped into “Others.” **E**, **F** Metabolic functional groups of endophytic communities of tree species identity and tree types, respectively. Blue and red circles indicate significant differences in the relative abundance of the taxonomic/functional groups among tree species and tree types, respectively (*P *<0.05, ANOVA for nine tree species; T-test for tree types)

The taxonomic compositions of endophytic bacterial communities varied at the phylum level among tree species and tree types (Fig. 1A). The relative abundance of Bacteroidota in the broadleaved group was higher than that in the coniferous group (*P* < 0.05), while the relative abundance of Proteobacteria in coniferous trees exhibited relatively lower than that in broadleaved trees (Fig. 1B). Additionally, the higher relative abundance of Proteobacteria was detected in the senesced needles of DG, KI, and LA tree species compared with others (Fig. 1A). From 1,219 endophytic bacterial ASVs, 35 functional groups related to C, N, and S cycling were identified using the FAPROTAX database (Fig. 1C). The relative abundance of bacteria primarily carrying out ureolysis (including *Massilia*) showed a significant increase in broadleaved trees compared to coniferous trees (Fig. 1D; Supplementary Data). The highest relative abundance of ureolysis was observed in the senesced leaves of HBU tree species (Fig. 1C), while the lowest relative abundance of ureolysis was observed in the senesced leaves of KB tree species. The relative abundance associated with hydrocarbon degradation, methylotrophy, methanotrophy, and N_2_ fixation in the coniferous group were higher than those in broadleaved tree species (Fig. 1D). There were significant differences in the relative abundances of all bacterial metabolic groups across tree species, as well as between tree type (Fig. 1E and F). Moreover, higher relative abundances of carbohydrate metabolism were detected in coniferous trees (Supplementary Fig. S3A, Supplementary Fig. S3B).

**Fig. 2:**
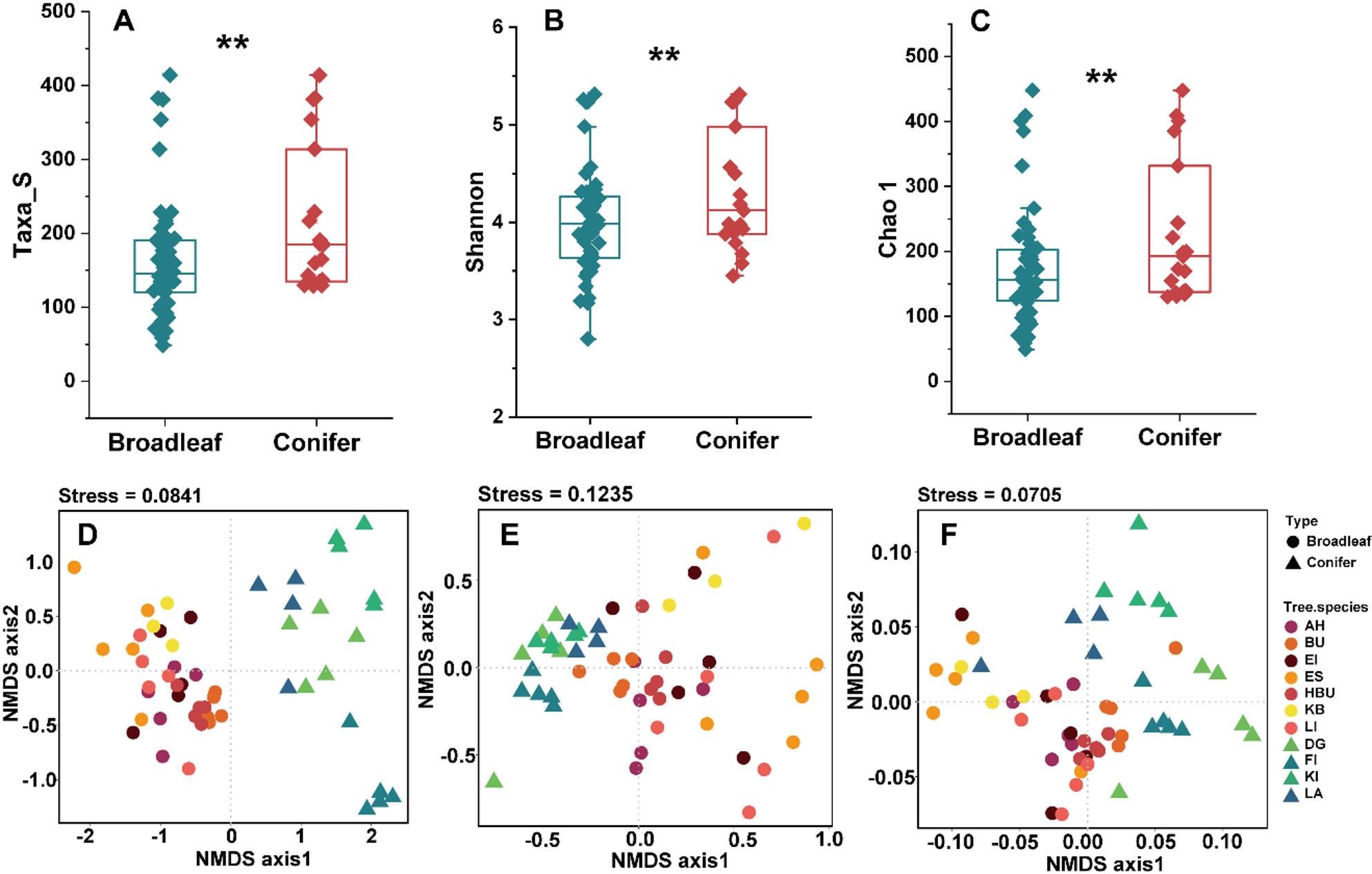
Endophytic bacterial alpha diversity indices (**A**–**C**) and nonmetric multidimensional scaling (NMDS) ordinations for taxonomic (**D**), ecological (**E**), and metabolic (**F**) compositions based on Bray-Curtis distance matrices between tree types. **, *P *<0.01

The α diversity indices were significantly higher in coniferous trees compared to broadleaved trees (Fig.2A–C). The variation in endophytic bacterial community composition was primarily explained by tree species identity (for taxonomy, *R*^*2*^ = 68.2%, *P* < 0.001; for ecological functions, *R*^*2*^ = 74.1%, *P* < 0.001; for metabolic function, *R*^*2*^ = 67.9%, *P* < 0.001, Supplementary Table S2).

### Endophytic bacterial co-occurrence network varied between tree types

**Fig. 3:**
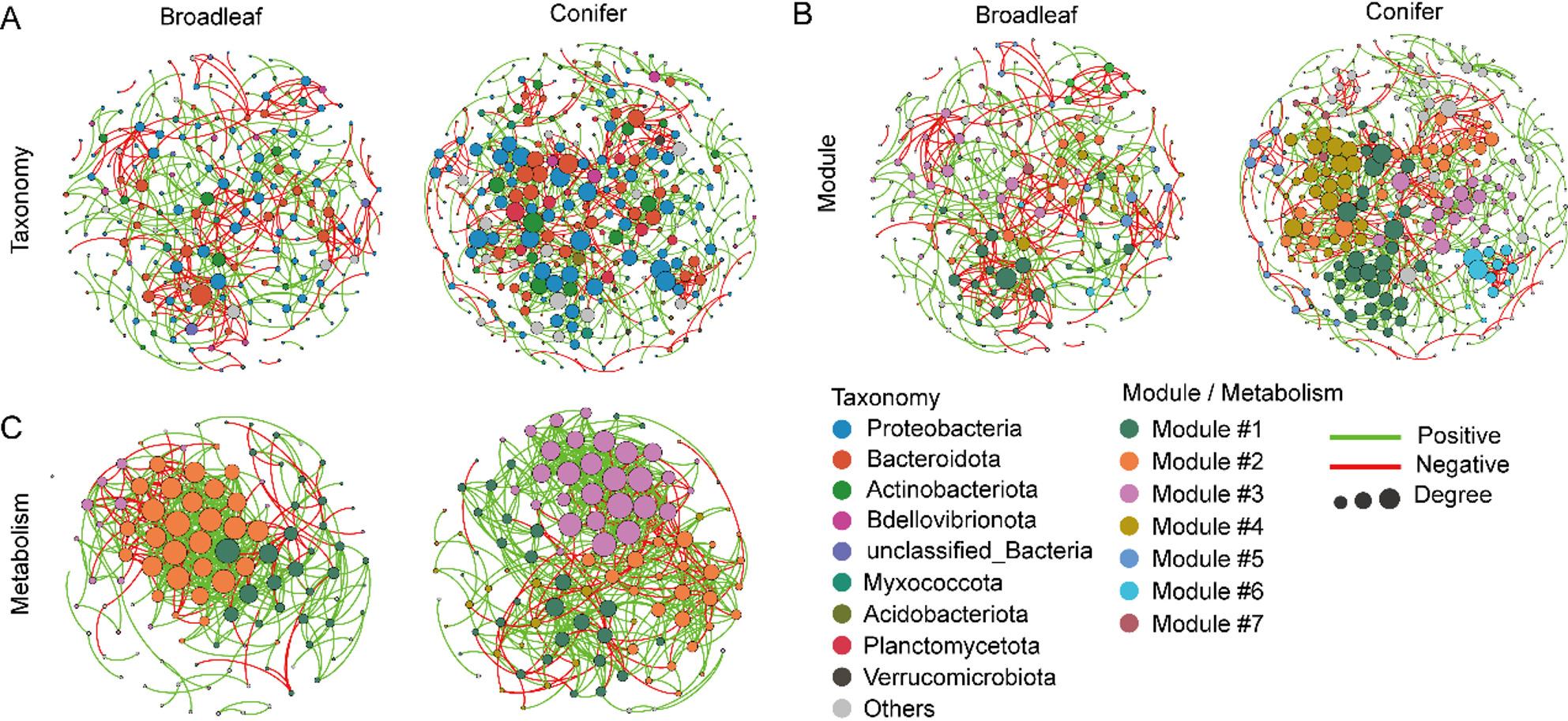
Taxonomic (**A**), modular (**B**), and metabolic networks (**C**) of endophytic bacteria between tree types. Node colors represent different phyla (**A**) and modules (**B**, **C**), respectively. The connections denote a strong (Spearman’s *ρ* > 0.6) and significant correlations (*P* < 0.01)

The taxonomic compositions of endophytic bacterial networks varied between the tree types (Table 1; Fig. 3). Compared to broadleaved trees, the bacterial network of coniferous trees contained more links, a higher average degree, and shorter average path distance (Fig. 3), which indicated that endophytic bacteria in the coniferous network were more sensitive to environmental fluctuations (Table 1). The higher average clustering coefficient showed that the bacterial network of the coniferous tree type was more connected than that of the broadleaved tree type. Similar patterns were also found in the metabolic network of endophytic bacteria (Fig. 3C, Supplementary Table S3). The complexity (higher average degree and shorter average path distance) of the taxonomic and metabolic network in the coniferous tree type was lower than that in the broadleaved tree type (Fig. 3B and C). Notably, the coniferous tree-based network posed a higher modularity than the modularity of broadleaved trees. The keystone species of network in broadleaved trees (3 module hubs and 3 connectors) was strikingly different from networks in coniferous trees (4 module hubs and 4 connectors, Supplementary Fig. S4, Supplementary Table S4).

**Table 1:** Topological properties of the taxonomic network in endophytic bacterial community composition

Network	Network features	Broadleaf	Conifer
Empirical network	Number of nodes	235	348
	Number of links	404	696
	R^2^ of power-law	0.826	0.863
	Number of positive correlations	243 (60.1%)	498 (71.6%)
	Number of negative correlations	161 (39.9%)	198 (28.4%)
	Average degree	3.438	4.000
	Average clustering coefficient	0.138	0.248
	Average path distance	5.907	5.834
	Modularity	0.734	0.784
Random network	Average clustering coefficient ± SD	0.017 ± 0.006	0.017 ± 0.004
	Average path distance ± SD	4.255 ± 0.066	4.088 ± 0.038
	Modularity ± SD	0.549 ± 0.009	0.500 ± 0.007

*SD* Standard deviation from XY replicates

### Stochasticity and determinism of endophytic assembly processes in mature leaves

**Fig. 4:**
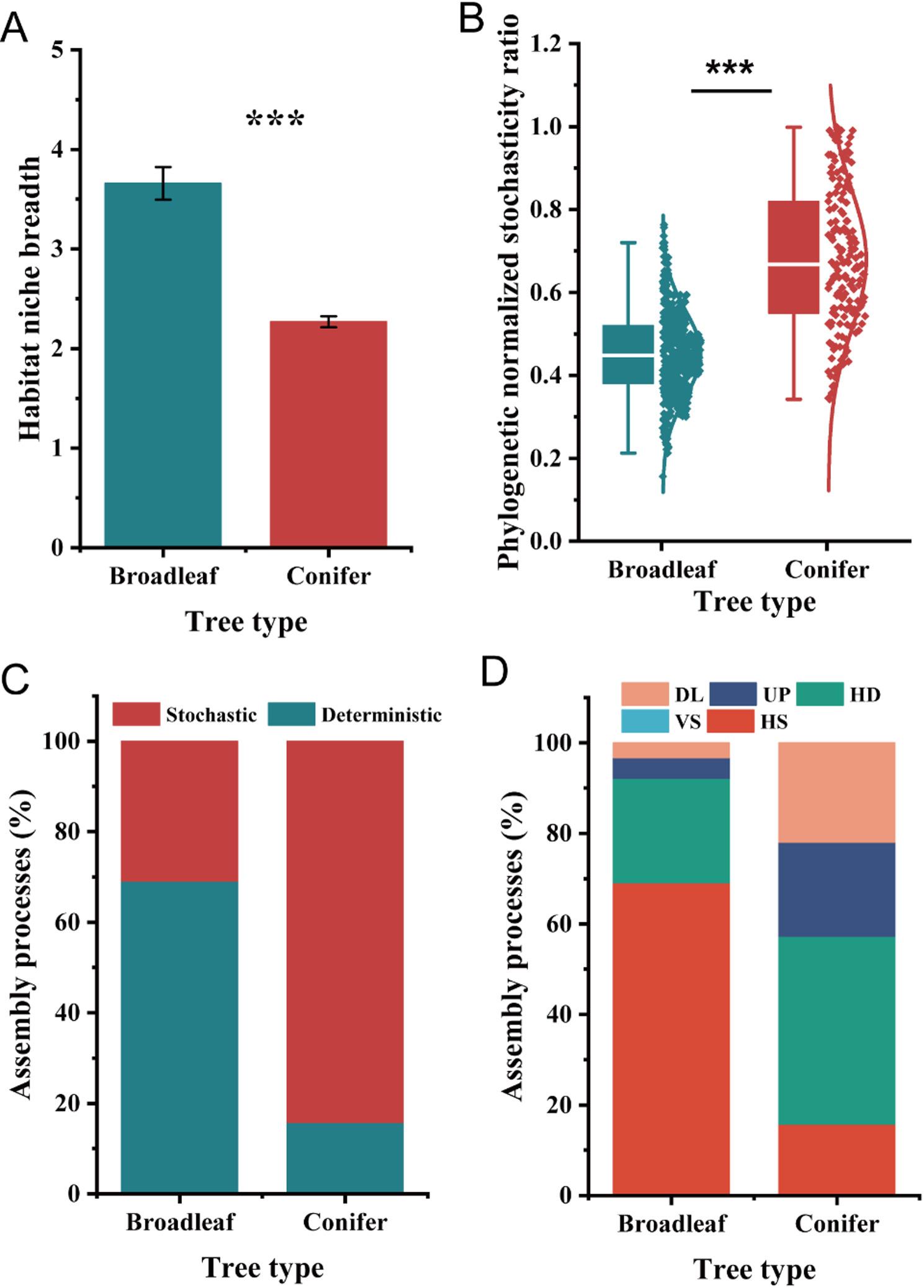
Habitat niche breadth (**A**) from all taxa (based on a 97% sequence similarity) in each sample of endophytic bacterial community composition. Significant differences between broadleaved and coniferous tree species are denoted with asterisks (*, *P *<0.05; ***, *P *<0.001). The ecological stochasticity in the potentially endophytic bacterial community assembly is estimated by the phylogenetic normalized stochasticity ratio (pNST) (**B**). The value of 0.5 is the boundary point between more deterministic (<0.5) and more stochastic (>0.5) assembly. Differences between broadleaf and coniferous tree species were examined using a *T*-test (***, *P *<0.001). The relative contributions (%) of the community assembly processes based on pNST (**C**) and βNTI (**D**) in shaping endophytic communities. HS, homogeneous selection; VS, variable selection; HD, homogenizing dispersal; UP, undominated process; DL, dispersal limitation

Based on Sloan’s neutral community model, the importance of stochasticity in endophytic assembly was analyzed. The model fit varied between tree type (broadleaved (*R*^*2*^ = 0.789), coniferous (*R*^*2*^ = 0.618)) as well as all tree species identity (*R*^*2*^ = 0.681, Supplementary Fig. S5A–C). Regarding community-level habitat niche breadth, a significant difference was observed among tree species identities (Supplementary Fig. S5D). The habitat niche breadth of endophytic bacterial communities in broadleaved trees was significantly higher than that in coniferous tree type (Fig. 4A). Additionally, the generalists and specialists were classified when the niche breadth value was higher or lower than simulated by chance, respectively (Fig. 4A). The endophytic bacterial communities comprised 25.8% of generalists and 47.5% of specialists in broadleaved trees and 31.1% of generalists and 41.5% of specialists in coniferous trees (Supplementary Fig. S5E). The proportions of both generalists and specialists demonstrated a slight difference among tree species (Supplementary Fig. S5F).

To evaluate and quantify ecological processes and selection, the phylogenetic normalized stochasticity ratio (pNST) was calculated based on null model analysis. The null model showed that the endophytic bacterial communities in broadleaved trees were mediated by deterministic processes whereas the communities in coniferous trees were primarily controlled by stochastic processes (Fig. 4B, C). The pNST values in coniferous trees were significantly higher than those in broadleaved trees (*P* < 0.001, Fig. 4B).

In addition, the endophytic bacterial community assembly in broadleaved and coniferous trees was predominantly controlled by homogeneous selection and homogenizing dispersal, respectively (Fig. 4D). The homogenizing dispersal of bacterial community in coniferous trees (41.5%) was higher than that in broadleaved trees (23.1%), respectively (Fig. 4D). Additionally, there was observed higher drift process in coniferous trees (20.8%) than that in broadleaved trees (4.6%). Environmental filtering acts as a deterministic selection process (specifically homogeneous selection) that influences the bacterial community assembly in broadleaved trees, while increased stochasticity was responsible for the bacterial community assemblages in coniferous trees.

### Potential associations between endophytic bacterial community and leaf properties

The bacterial community composition in broadleaved trees was primarily affected by leaf NH_4_ and N_min_ concentrations, while the geographical locations (latitude and longitude) showed a strong association with the endophytic community composition in conifers (*R*^*2*^_latitude_=0.88, *R*^*2*^_longitude_=0.92) (Table 2). In addition, all endophytic bacterial communities in conifers exhibited a strong correlation with DOC, Ca, and P contents (Table 2).

**Table 2:** Goodness-of-fit statistics (*R*^2^) of environmental variables with nonmetric multidimensional scaling (NMDS) ordination of endophytic bacterial taxonomic composition based on abundance data and Bray-Curtis distance measure

Leaf variables	Broadleaved and conifer tree species	Broadleaved trees	Conifer trees
Leaf water content (%)	0.18**	0.01	0.16
DOC (mg/g dry weight)	0.60***	0.06	0.47**
DOC_richness	0.66***	0.12	0.47**
NH_4_ (mg/g dry weight)	0.40***	0.48***	0.05
NO_2_ (mg/g dry weight)	0.43***	0.01	0.08
N_min_ (mg/g dry weight)	0.35***	0.30**	0.04
N_org_ (mg/g dry weight)	0.56***	0.13	0.06
TN (mg/g dry weight)	0.56***	0.15	0.05
Ca (mg/g)	0.65***	0.16	0.36*
Fe (mg/g)	0.27**	0.16	0.08
K (mg/g)	0.01	0.03	0.04
Mg (mg/g)	0.51***	0.20*	0.22
P (mg/g)	0.34***	0.12	0.38*
pH	0.14*	0.45***	0.57**
Latitude	0.64***	0.04	0.82***
Longitude	0.15*	0.17	0.66***

*DOC* Dissolved organic carbon, *NH*_4_, ammonium nitrogen, *N*_min_, mineral nitrogen, *N*_org_, organic nitrogen, *TN* Total nitrogen;

All significant *R*^*2*^ values are highlighted in bold. *, *P* < 0.05; **, *P* < 0.01; ***, *P* < 0.001

**Fig. 5:**
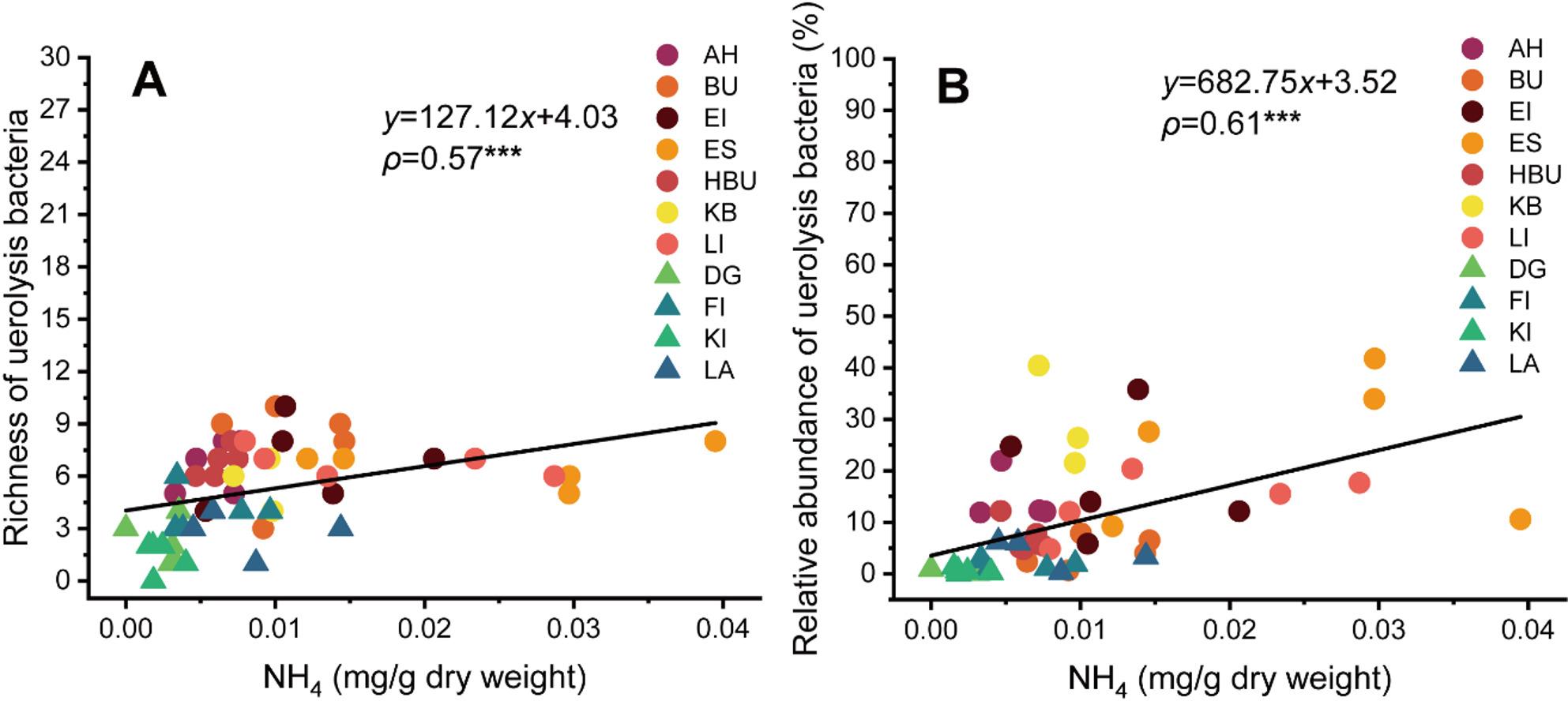
Relationships between NH4 and ureolytic bacterial richness (**A**) and relative abundance (**B**) across eleven tree species. The circle and triangle symbols represent the broadleaved and coniferous trees, respectively. The black line represents the fitted linear regression curve. ***, *P *<0.001

All leaf variables related to N fractions strikingly affected bacterial community functions in broadleaved trees. Specifically, ureolytic bacteria in broadleaved trees had stronger associations with leaf variables than that in coniferous trees. Moreover, ureolytic bacteria were significantly affected by N fractions (excluding NO_2_) in broadleaved trees (Supplementary Table S5). The content of NH_4_ was positively correlated with the richness (*ρ* = 0.57, *P* < 0.001) and relative abundance (*ρ* = 0.61, *P* < 0.001) of ureolytic bacteria (Fig. 5A, B). The results of the partial Mantel test showed that the amino acid function of endophytic bacteria had strong associations with NH_4_ content, pH, P content, latitude, and longitude (Supplementary Fig. S6).

## Discussion

### Deterministic processes determine bacterial communities of senescing leaves in broadleaved trees: fall preparation

Plants can recruit bacterial communities and are collectively defined as the holobiont [11, 60], and the community assembly, structure, and specific functional taxa of endophytic bacteria become important for early-stage litter decay during the leaf maturation stage [61]. In general, due to the physiological difference of leaf form related to nutrients, coniferous litter with higher C: N and lignin: N exhibit slower decomposition rates than broadleaved litter, especially in N-limited temperate forest ecosystems [36]. The results of this study showed that specific endophytic taxa (ureolytic taxa) inhabiting leaves and providing N in broadleaved trees may play more important roles just before the leaves fall. Our subset analysis of ureolytic bacterial diversity showed a significant correlation with N nutrients (NH_4_ and N_min_) in broadleaved trees (Supplementary Table S5), indicating that the major form of mineralized N is NH_4_, which makes sense as NH_4_ is the product of ureolysis [62]. The high inorganic N in leaves of broadleaved trees may not only come from the tree host, inorganic N is actually contributed by ureolytic bacteria which generate urea from organic sources (proteins, chitin, and amino acid) at leave maturation or senescence [62]. Leaves (e.g. broadleaved) with low C: N, high nutrient content, and low amounts of recalcitrant compounds (e.g., lignin and tannins) can be microbially decomposed more rapidly [63]. In addition, the leaves of coniferous trees (e.g., DG and LA) have a relatively narrow habitat niche breadth compared with that of broadleaved trees (Fig. 4), indicating that broadleaved trees provide abundant resources for endophytic bacteria. The different assemblages of endophytic bacteria between broadleaved and coniferous trees may be the result of these varied strategies of leaf form.

Revealing the process and mechanism of community assembly of endophytic bacteria is essential for understanding ecosystem services and functions [23]. As expected, the endophytic bacterial communities in broadleaved trees were primarily governed by a deterministic process and were less affected by geospatial factors. Moreover, the dispersal limitation and drift processes were less impactful than those in coniferous trees, respectively (Fig. 4). These findings exhibited an inclination toward minimized spatial variation, which is in line with the findings of Wang et al. [64], based on the estimated result by null model analysis, the homogenizing selection was predicted to become the dominant role in broadleaved trees. As far as we know, this is the first report regarding the community assembly of endophytic bacteria. Despite the mechanisms underlying community assembly of endophytic bacteria across host trees were rarely addressed in the literature, increasing empirical evidence demonstrated the bacterial community tend to mediate by determinism in soil [65] and aquatic ecosystems [66]. Taken together, our results demonstrate that the distinct patterns of bacterial community assembly and functional potential characteristic of broadleaved trees, relative to those in coniferous trees, lead to a greater potential for accelerated leaf decomposition.

### Slow leaf degradation from coniferous trees acquires a critical decomposer composition

The nutrient status of leaves following senescence affects litter quality and decomposition rates and also plays a pivotal role in the nutrient cycling of terrestrial ecosystems [67]. Despite the importance of leaves, there remains a paucity of evidence on the nutrient acquisition of endophytic microbiota compared with root-associated microbiota. The endophytic community in coniferous trees showed a greater habitat niche breadth than that in broadleaved trees (Fig. 4), which indicates less bio-available nutrient resources in coniferous needles. Moreover, the ecological functions of endophytes in coniferous trees (especially in FI tree species) were more diverse compared to broadleaved trees (Fig. 1D). This may be because needles, with an active nutrient acquisition strategy, exhibit more stable, closed, and specialized features [68] and can achieve nutrient transformation independently by recruiting specific taxa exert distinct functional properties. In addition, the greater percentage of negative links of the coniferous trees implied co-occurring taxa of bacteria in coniferous trees were more likely to compete with limited nutritional resources and niche partitioning (Table 1; Fig. 3A). Previous studies demonstrated that niche adaptation plays crucial roles in the selective filtering and recruitment of microorganisms [23]. Nonetheless, the recruitment must be interpreted with caution as this study was limited by a lack of information on fine-scale manipulation and real-time imaging of plant recruitment and microbial colonization in a microfluidics approach. Additionally, valuable insights into plant-microbe interactions, which potentially involve plant exudates, phytohormones, and internal signaling cascades, can be obtained through the use of multi-omics techniques.

Proteobacteria normally constitute ~ 50% of the total endophytic community composition [61] and can contribute to nitrification, N_2_ fixation, methylotrophy, or anoxygenic photosynthesis [15]. Actinobacteria include members of plant pathogens, N_2_-fixing symbionts, plant growth promoting [69] as well as fungal antagonistic capabilities [70]. The relative abundance of Proteobacteria in coniferous trees was significantly higher than that in broadleaved trees, and tree identity had a striking effect on the relative abundance of Actinobacteria (dramatically higher in Douglas fir and spruce) (Fig. 1), indicating the diverse functions of endophytic bacteria among temperate tree species. Netherway et al. [71] stated that needles perform less functionally redundant roles, leading to greater acidity and higher C: N in the habitat via direct selective pressure and mediating N acquisition and transformation. Interestingly, the endophytic N_2_-fixing bacterial communities were observed in a higher relative abundance in coniferous trees, respectively (Fig. 1). Fürnkranz et al. [15] found that cyanobacterial diversity, which includes many N_2_-fixing species, is highly variable on leaf surfaces depending on host tree species and environment conditions, and can provide significant N input for a rainforest ecosystem. This coincidental finding implies that senesced needles exhibit a contrasting strategy in endophytic community assembly and associated bacterial functions compared to leaves.

### Leaf physicochemical properties are not everything: stochasticity can be influential

The process of litter decay can be determined to some extent by the trajectory for the forthcoming microbiome (priority effects) in the assembly of the senesced leaf microbiome [61, 72]. Deciphering and quantifying the relative contribution of deterministic vs. stochastic processes is crucial to differentiating the origin, priming, and potential contribution to ecosystem services of endophytic communities [73]. Our results indicate that endophytic bacterial communities in broadleaved trees are primarily governed by deterministic processes, even at a local scale, though tree-specific stochastic processes are also anticipated. In contrast, coniferous trees exhibited a prevalence of homogenizing dispersal, potentially modulated by local environmental factors such as site-specific conditions. These findings were corroborated by our empirical network analyses as our networks showed higher complexity and higher robustness to stress from habitat conditions [74]. Networks of broadleaved trees were relatively small and simple, and exhibited increasing positive links compared to coniferous networks, which is suggestive of their vulnerability to environmental filter processes (deterministic selection). Hernandez et al. [75] demonstrated that persistent stress decreases the modularity, complexity, and stability of the microbial networks while increasing negative interactions among microbial taxa. More interestingly, the simple network in broadleaved trees suggests that their leaves (i) are more susceptible to stress, and (i) face interference from a more variable and hostile environment, which further supports the importance of selection in broadleaves. Recent studies illustrated that the stochastic processes dominate in high-diversity communities, whereas deterministic processes are more prevalent in low-diversity communities [76]. Our results align with these findings, indicating that α-diversity in coniferous trees is significantly higher compared to broadleaved trees (Fig. 2A–C). For different tree species identities, in combination with certain leaf physicochemical factors, specific bacterial taxa may be selected to form holobiont with distinct functional properties in preparation for decay.

## Conclusions

**Fig. 6:**
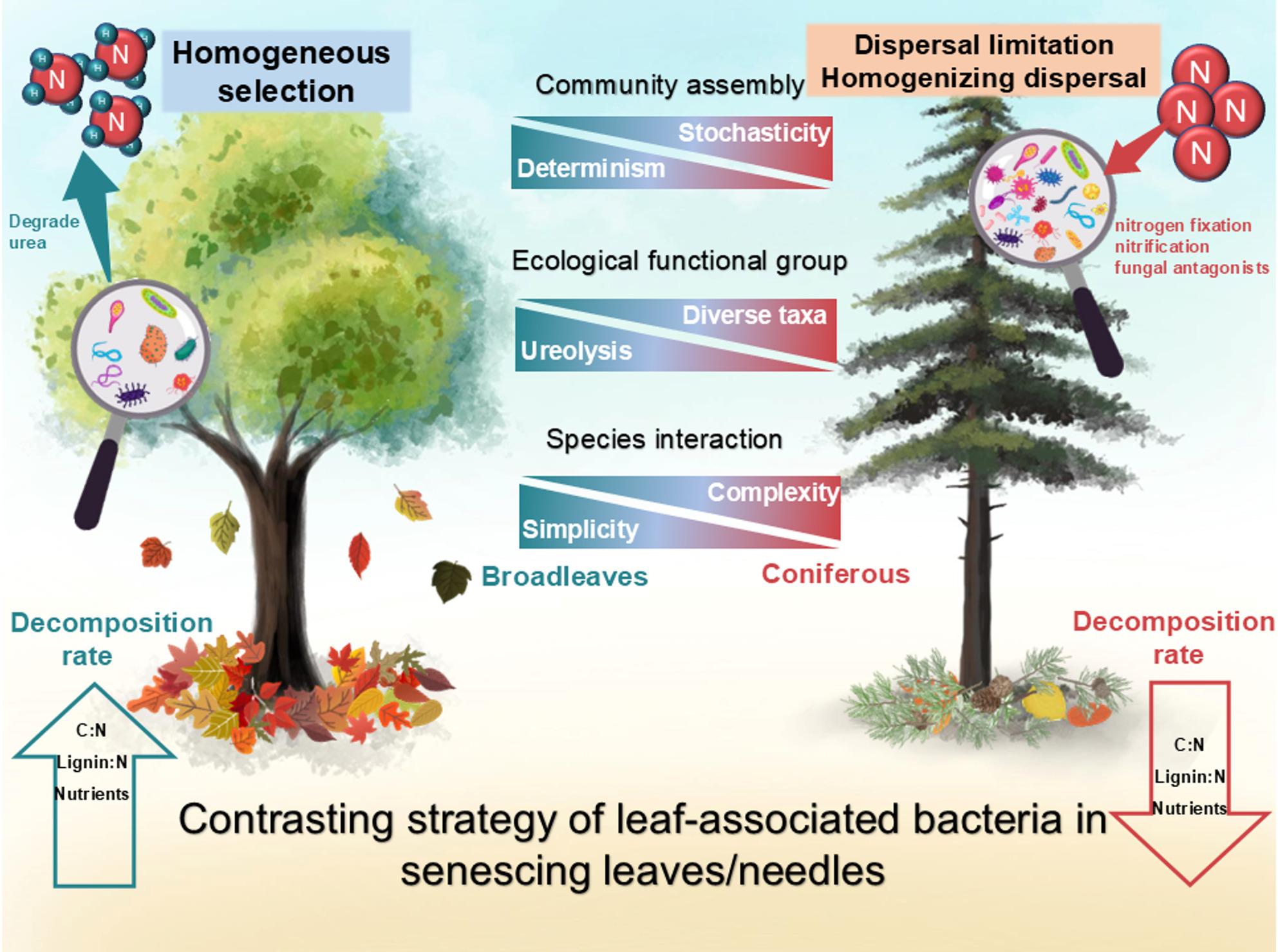
Conceptual diagram portraying the contrasting strategy of endophytic bacteria in senescing leaves/needles between broadleaf and coniferous trees. Briefly, endophytic bacteria in broadleaved tree species were predominantly mediated by determinism, while stochasticity was observed in coniferous tree species. The senescing leaves from broadleaves could colonize ureolytic bacteria to degrade ammonium, and the endophytic bacteria inhabiting needles had more diverse taxa to prepare the organic nutrient economy

In conclusion, by examining the endophytic bacterial community structure from senescing leaves, and tree types in Central European forests, this study proposes a conceptual paradigm for assessing the bacterial attributes and their link to physicochemical leaf/needle properties and decay rate (Fig. 6). Tree type had a significant effect on multiple endophytic bacterial community attributes (i.e., diversity, community structure, ecological and metabolic function, ecological stochasticity, and co-occurrence networks). Furthermore, leaves from broadleaved tree species were deterministically colonized by a defined set of taxa (i.e. ureolysis), which potentially contributes to the N availability enabling a subsequent litter degradation at the early stage of litter decomposition. In contrast, conifers were colonized by a broader diversity in a stochastic manner distinguishing strategy, which had highly diverse bacterial taxa inhabiting to maintain the nutrient acquisition. The network demonstrated cooperative and synergistic interactions within the senescing leaf bacterial community, supporting that endophytic bacteria in broadleaved tree species are vulnerable to environmental selection. Overall, our results underscore the significant role of tree type in elucidating the assembly mechanisms of endophytic communities in matured leaves and needles.

## Supplementary Information

Supplementary Material 1.

Supplementary Material 2.

## Data Availability

Next-generation sequencing data has been deposited in the National Center for Biotechnology Information (NCBI) Sequence Read Archive (SRA) database under BioProject ID: PRJNA753096.

## Abbreviations

C: Carbon
N_2_: Nitrogen
P: Phosphorus
ASVs: Amplicon sequence variants
*m*: migration rate
pNST: phylogenetic normalized stochasticity ratio
NMDS: Nonmetric multidimensional scaling
PERMANOVA: Permutation multivariate analysis of variance
NCM: Neutral community model
RC_bray_: Raup-Crick value based on Bray–Curtis distance
