# Supplementary material for: Tree type-specific endophytic bacterial assembly and function in senescing leaves and needles in temperate forests of Central Europe
Source: BMC Plant Biol. 2026 May 21;26:900. doi: 10.1186/s12870-026-08931-x (PMC13192065; doi:10.1186/s12870-026-08931-x)
Supplement: Supplementary file 1 — Supplementary Material 1. [file 12870_2026_8931_MOESM1_ESM.docx]

**Supplementary Material**

**Supplementary text (Measurement of leaf variables)**

**Physiochemical analyses**

To obtain the water-leachable components, the senescing leaf and needle samples were shaken in 30 mL milliQ water for 1 h in falcon tubes, centrifuged for 5 min at 3500 rpm, decanted, and filtered. The remaining leaf/needle material was dried for two weeks at 40 °C to determine dry weight which was used as a reference for all qualifications. The pH of the leachates was determined using pH paper with a scale precision of 0.2 pH units. TN was analyzed using a sum parameter analyzer with high temperature combustion and chemiluminescence detection (Mitsubishi TN-100; a1 envirosciences, Düsseldorf, Germany). All samples were measured as triplicates. N_org_ was calculated as the difference: N_org_ = TN – N_Min_. For N_Min_ quantification, a flow injection analyzer (Quikchem QC85S5; Lachat Instruments, Hach Company, Loveland CO, USA) with corresponding manifolds for the measurement of ammonium nitrogen $N_{\mathrm{NH}_{4}^{+}}$, nitrite nitrogen $N_{\mathrm{NO}_{2}^{-}}$, and nitrate- plus nitrite nitrogen $N_{\mathrm{NO}_{3}^{-}+\mathrm{NO}_{2}^{-}}$ was used. $N_{\mathrm{NH}_{4}^{+}}$ was determined by the gas diffusion method. $N_{\mathrm{NO}_{3}^{-}}$ was reduced to nitrite using a cadmium column in the manifold prior to the chemical reaction to form an azo dye. The nitrate reduced by cadmium and the nitrite originally present in the sample were analyzed using the Griess reaction by diazotization with sulfanilamide and coupling with N-(1-naphthyl) ethylenediamine dihydrochloride. The deep pink color of the resulting dye was measured at λ = 520 nm. $N_{\mathrm{NO}_{2}^{-}}$ alone was determined after the same reaction, without using a cadmium column. DOC was quantified as non-purgeable organic carbon (NPOC) with a sum parameter analyzer using high-temperature combustion and infrared detection (vario TOC cube, Elementar Analysensysteme GmbH, Langenselbold, Germany). Each sample was measured in triplicate independent measurements. A sample volume of 200 µL each was automatically injected into the ash finger of the combustion tube which contains platinum as catalyst. The samples were combusted at 850°C in synthetic air, a hydrocarbon-free mixture of N and O_2_. After removing moisture from the combustion gas, NPOC was quantified by IR detection of CO_2_ formed from the organic carbon compounds in the sample.

The determination of nutrient content, Ca, Fe, K, Mg, and P of leaves and needles followed two processes. First, the sample digestion, in which 100 mg of sample material was submitted to a microwave-assisted high-pressure digestion (Multiwave 3000, Anton Paar, Graz, Austria) at a maximum microwave power of 1200 W and a maximum pressure of 60 bar after addition of 3 – 5 mL 65% HNO_3_, supra-pur, (Merck, Darmstadt, Germany). A rotor 8SXF100 with reaction vessels made of TFM (tetrafluor-modified polytetrafluoroethylene) was used. Overall digestion time was 50 min, including 20 min of cooling at zero microwave power. A blank, consisting of nitric acid only was run to check for possible contamination of reagents and vessels. After accomplishment of digestion, the resulting solutions were filtered and transferred to 50 mL PE vessels, which were filled to the mark with ultrapure water (Millipore, Eschborn, Germany). Secondly, the sample solution analyses were carried out by Inductively Coupled Plasma–Optical Emission Spectrometry (ICP-OES) “Arcos” (Spectro, Kleve, Germany) equipped with a 27.12 MHz free-running LDMOS generator and ORCA optical system. A three-point-calibration based on single-element standards issued by Merck, Darmstadt, Germany, was carried out at the following concentration levels: 10, 50, 100 mg/L for Ca, K, Mg, P and 0.5, 2.5 and 5 mg/L for Fe, respectively.

**DNA extraction, Illumina sequencing, and bioinformatics**

Mature leaves and needles, healthy leaves (up to 10 leaves per tree individual, depending on leaf size), and needles (from five branches per tree individual) were subsampled and prepared for DNA extraction. The leaf and needle samples were washed and DNA was extracted using the DNeasy PowerSoil Kit (Qiagen, Hilden, Germany). The presence and amount of DNA were checked using a NanoDrop ND-1000 spectrophotometer (Thermo Fisher Scientific, Dreieich, Germany), and the extracts were stored at −20 °C.

For the library preparation, the polymerase chain reactions (PCR) were performed using 20 μL reaction volumes with 5× HOT FIRE Pol Blend Master Mix (Solis BioDyne, Tartu, Estonia) on ABI Veriti thermocyclers (Applied Biosystems, Carlsbad, CA, USA). The bacterial polymerase chain reaction (PCR) conditions were as follows:95 °C for 15 min, followed by 30 cycles of 95 °C for 20 s, 56 °C for 30 s, 72 °C for 1 min, followed by one extension cycle at 72 °C for 5 min, and a 4 °C hold. The amplified products were visualized using gel electrophoresis. Amplification products (three replicate reactions per sample) were pooled in equimolar amounts and purified using an Agencourt AMPure XP kit (Beckman Coulter, Krefeld, Germany). Illumina Nextera XT Indices were added at both ends of the fungal amplicons. Paired-end sequencing (2 × 300 bp) was performed at the Department of Soil Ecology, Helmholtz Centre for Environmental Research, Germany.

**Supplementary Table S1** Tree species and their abbreviated names in this study

| Tree species | Abbreviation | Family | Tree type |
| --- | --- | --- | --- |
| *Acer pseudoplatanus* | Ah | Sapindacea | Broadleaf |
| *Fagus sylvatica* | Bu | Fagaceae | Broadleaf |
| *Quercus robur* | Ei | Fagaceae | Broadleaf |
| *Fraxinus excelsior* | Es | Oleaceae | Broadleaf |
| *Carpinus betulus* | Hbu | Betulaceae | Broadleaf |
| *Prunus avium* | Kb | Rosaceae | Broadleaf |
| *Tilia cordata* | Li | [Malvaceae](http://www.cfh.ac.cn/815.sp) | Broadleaf |
| *Pseudotsuga menziesii* | Dg | Pinaceae | Conifer |
| *Picea abies* | Fi | Pinaceae | Conifer |
| *Pinus sylvestris* | Ki | Pinaceae | Conifer |
| *Larix decidua* | La | Pinaceae | Conifer |

**Supplementary Table S2** Effects of tree species, tree type and mycorrhizal association on the compositional differences of endophytic bacterial communities based on PERMANOVA with 999 permutations.

|  | Taxonimic composition | | | | Ecological function | | | | Metabolic function | | | |
| --- | --- | --- | --- | --- | --- | --- | --- | --- | --- | --- | --- | --- |
|  | df | *F* | *R^2^* | Pr (>*F*) | df | *F* | *R^2^* | Pr (>*F*) | df | *F* | *R^2^* | Pr (>*F*) |
| Tree species | 10 | 8.811 | 68.246 | **0.001** | 10 | 11.731 | 74.101 | **0.001** | 10 | 8.655 | 67.855 | **0.001** |
| Tree type | 1 | 26.731 | 34.838 | **0.001** | 1 | 27.106 | 46.205 | **0.001** | 1 | 18.790 | 27.315 | **0.001** |

All significant *P* values are highlighted in bold followed by significance level codes.

**Supplementary Table S3** Topological properties of metabolic network in endophytic bacteria

| Network | Network features | Broadleaf | Conifer |
| --- | --- | --- | --- |
| Empirical network | Number of nodes | 109 | 114 |
|  | Number of links | 507 | 561 |
|  | *R*^2^ of power-law | 0.583 | 0.534 |
|  | Number of positive correlations | 413 (81.5%) | 451 (80.4%) |
|  | Number of negative correlations | 94 (18.5%) | 110 (19.6%) |
|  | Average degree (avgK) | 9.303 | 9.842 |
|  | Average clustering coefficient (avgCC) | 0.457 | 0.513 |
|  | Average path distance (GD) | 3.591 | 3.392 |
|  | Modularity | 0.342 | 0.515 |
| Random network | avgCC±SD | 0.239±0.017 | 0.169±0.013 |
|  | GD±SD | 2.558±0.050 | 2.444±0.031 |
|  | Modularity±SD | 0.213±0.007 | 0.230±0.007 |

**Supplementary Table S4** Topological roles of ASVs in the endophytic bacterial networks

|  | ASV ID | Topological role | Function | Phylum | Class | Order | Family | Genus |
| --- | --- | --- | --- | --- | --- | --- | --- | --- |
| Broadleaf | ASV_001062 | Module hub | chemoheterotrophy | Pseudomonadota | Alphaproteobacteria | Sphingomonadales | Sphingomonadaceae | *Sphingomonas* |
|  | ASV_000437 | Module hub | chemoheterotrophy | Actinobacteriota | Actinobacteria | Kineosporiales | Kineosporiaceae | *Kineococcus* |
|  | ASV_000996 | Module hub | —— | Bacteroidota | Bacteroidia | Cytophagales | Hymenobacteraceae | *Hymenobacter* |
|  | ASV_000034 | Connector | aerobic_chemoheterotrophy | Pseudomonadota | Alphaproteobacteria | Sphingomonadales | Sphingomonadaceae | *Sphingomonas* |
|  | ASV_001260 | Connector | —— | Bacteroidota | Bacteroidia | Cytophagales | Spirosomaceae | *Dyadobacter* |
|  | ASV_000609 | Connector | —— | Myxococcota | Myxococcia | Myxococcales | Myxococcaceae | P3OB-42 |
|  |  |  |  |  |  |  |  |  |
| Conifer | ASV_000741 | Module hub | —— | Pseudomonadota | Alphaproteobacteria | Rhizobiales | Beijerinckiaceae | 1174-901-12 |
|  | ASV_001348 | Module hub | —— | Pseudomonadota | Alphaproteobacteria | Micavibrionales | uncultured | uncultured_unclassified |
|  | ASV_001039 | Module hub | —— | Pseudomonadota | Alphaproteobacteria | Rhizobiales | Beijerinckiaceae | *Psychroglaciecola* |
|  | ASV_001386 | Module hub | —— | Bacteroidota | Bacteroidia | Sphingobacteriales | Sphingobacteriaceae | *Mucilaginibacter* |
|  | ASV_000179 | Connector | chemoheterotrophy | Actinobacteriota | Actinobacteria | Corynebacteriales | Mycobacteriaceae | *Mycobacterium* |
|  | ASV_000224 | Connector | —— | Planctomycetota | Planctomycetes | Isosphaerales | Isosphaeraceae | Isosphaeraceae_unclassified |
|  | ASV_000390 | Connector | —— | Bacteroidota | Bacteroidia | Chitinophagales | Chitinophagaceae | *Heliimonas* |
|  | ASV_000677 | Connector | aerobic_chemoheterotrophy | Pseudomonadota | Alphaproteobacteria | Sphingomonadales | Sphingomonadaceae | *Sphingomonas* |

**Supplementary Table S5** Goodness-of-fit statistics (*R*^2^) of environmental variables fitted to the nonmetric multidimensional scaling (NMDS) ordination of ecological function derived from endophytic bacterial diversity and Bray–Curtis distance measure.

| Leaf variables | All species | | Broadleaf | | Conifer | |
| --- | --- | --- | --- | --- | --- | --- |
|  | Ecological functions | Ureolytic diversity | Ecological functions | Ureolytic diversity | Ecological functions | Ureolytic diversity |
| Leaf water content (%) | **0.16*** | 0.06 | **0.22*** | 0.08 | 0.28 | **0.36*** |
| DOC (mg/g dry weight) | **0.55***** | 0.11 | **0.28**** | **0.28**** | **0.35*** | 0.26 |
| DOC_richness | **0.35***** | **0.23**** | 0.03 | 0.02 | 0.19 | 0.15 |
| NH_4_ (mg/g dry weight) | **0.44***** | 0.1 | **0.37**** | **0.35**** | 0.08 | 0.31 |
| NO_2_ (mg/g dry weight) | **0.44***** | 0.06 | **0.23*** | 0.14 | 0.26 | **0.51**** |
| N_min_ (mg/g dry weight) | **0.34***** | **0.12*** | **0.18*** | **0.28*** | 0.08 | 0.31 |
| N_org_ (mg/g dry weight) | **0.50***** | **0.13*** | **0.21*** | **0.38***** | 0.14 | **0.47*** |
| TN (mg/g dry weight) | **0.50***** | **0.13*** | **0.20*** | **0.39***** | 0.13 | 0.05 |
| Ca (mg/g) | **0.36***** | **0.17**** | 0.03 | 0.08 | 0.14 | 0.06 |
| Fe (mg/g) | 0.08 | 0.05 | 0.09 | **0.21*** | 0.07 | 0.22 |
| K (mg/g) | 0.01 | 0.01 | **0.24*** | 0.07 | 0.13 | 0.15 |
| Mg (mg/g) | **0.52***** | **0.12*** | **0.23*** | 0.17 | 0.28 | **0.36*** |
| P (mg/g) | **0.35***** | 0.12 | 0.10 | 0.05 | **0.33*** | 0.02 |
| pH | **0.25**** | 0.02 | **0.39**** | **0.45***** | **0.44*** | 0.06 |
| Latitude | **0.47***** | 0.08 | 0.06 | 0.01 | 0.08 | **0.28*** |
| Longitude | 0.16* | 0.04 | 0.12 | 0.14 | 0.01 | **0.39*** |

DOC, dissolved organic carbon; NH_4_, ammonium nitrogen; N_min_, mineral nitrogen; N_org_, organic nitrogen; TN, total nitrogen;

All significant *R^2^* values are highlighted in bold. *****, *P*<0.05; ******, *P*<0.01; *******, *P*<0.001


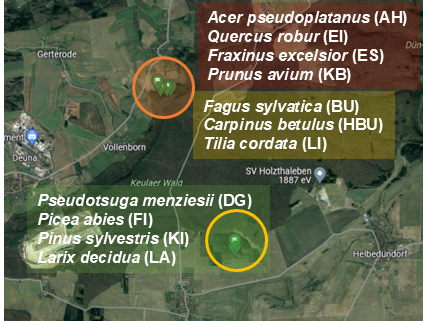


**Supplementary Fig. S1** The eleven temperate tree species located in the Hainich-Dün region of Thuringia, Germany (51°12’N 10°18’E). The map is modified from a previous study (More than you can see: <https://doi.org/10.3389/fmicb.2022.907531>). Source: Google Maps, 2022. Hainich-Dün region of Thuringia. Google Maps [online] Available at: https://www.google.com/maps, [Accessed 12 July 2022]

**
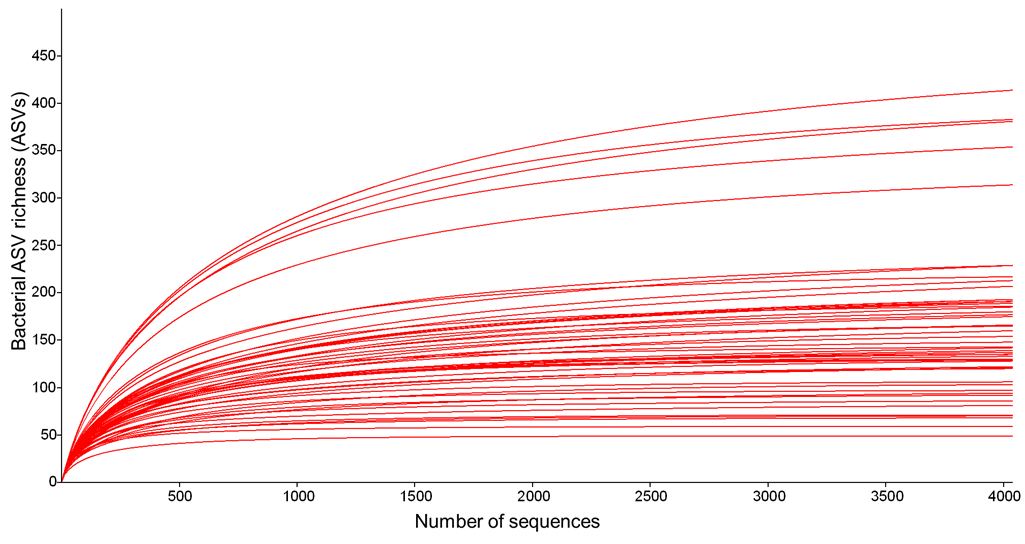
**

**Supplementary Fig. S2** Rarefaction curves of the ASV number of each endophytic bacterial community of nine temperate tree species.


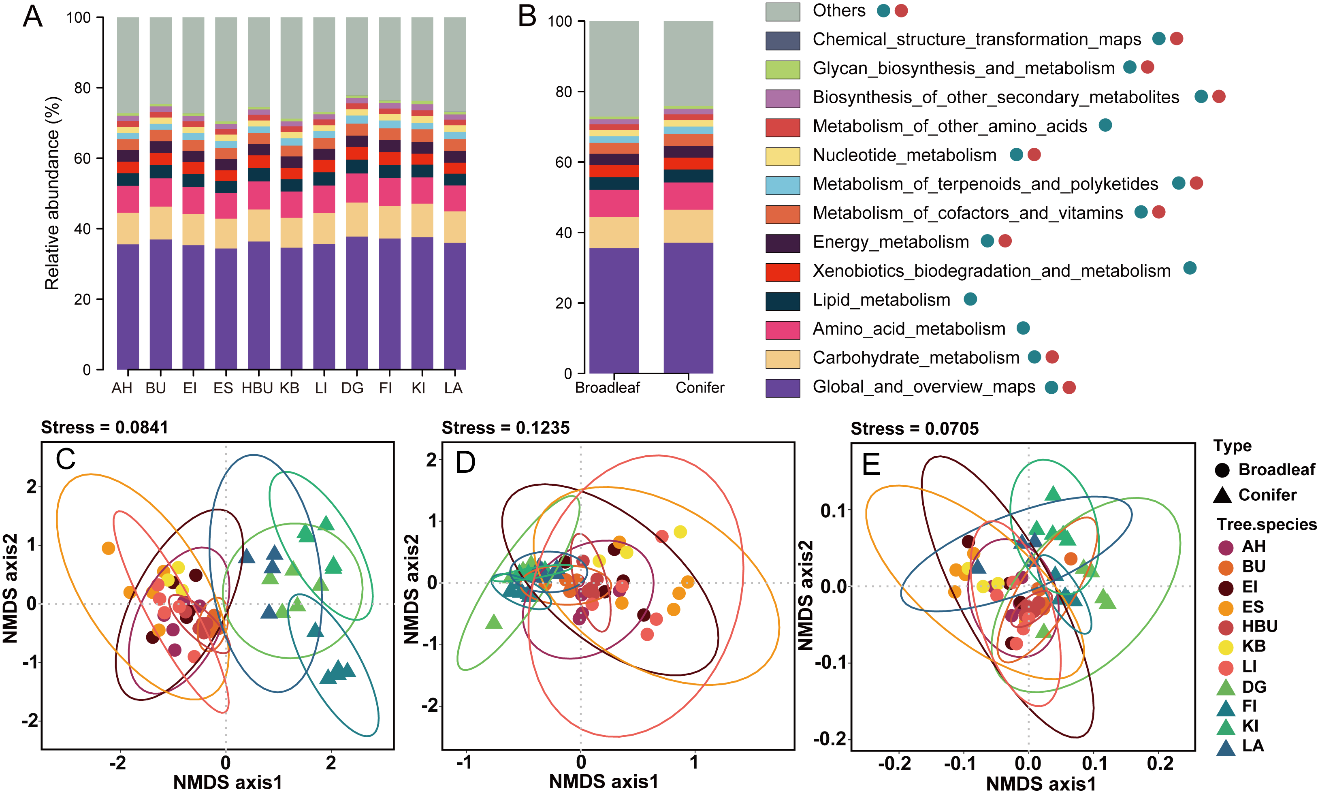


**Supplementary Fig. S3** Functional composition for metabolism (Level 2) of endophytic bacterial communities in nine temperate tree species in Central European forests. (A, B), metabolic functional groups of endophytic bacterial communities of tree species, and tree types, respectively. Nonmetric multidimensional scaling (NMDS) ordinations for taxonomic (C), ecological (D) and metabolic (E) compositions based on Bray-Curtis distances matrices of endophytic communities for nine tree species. Ellipses represent 95% confidence intervals around tree-type centroids. The top 13 most abundant functional groups for metabolism were shown. Blue and red circles indicate significant differences in the relative abundance of the functional groups for metabolism among tree species or between tree types, respectively (*P*<0.05, ANOVA for nine tree species; *T-*test for tree types).


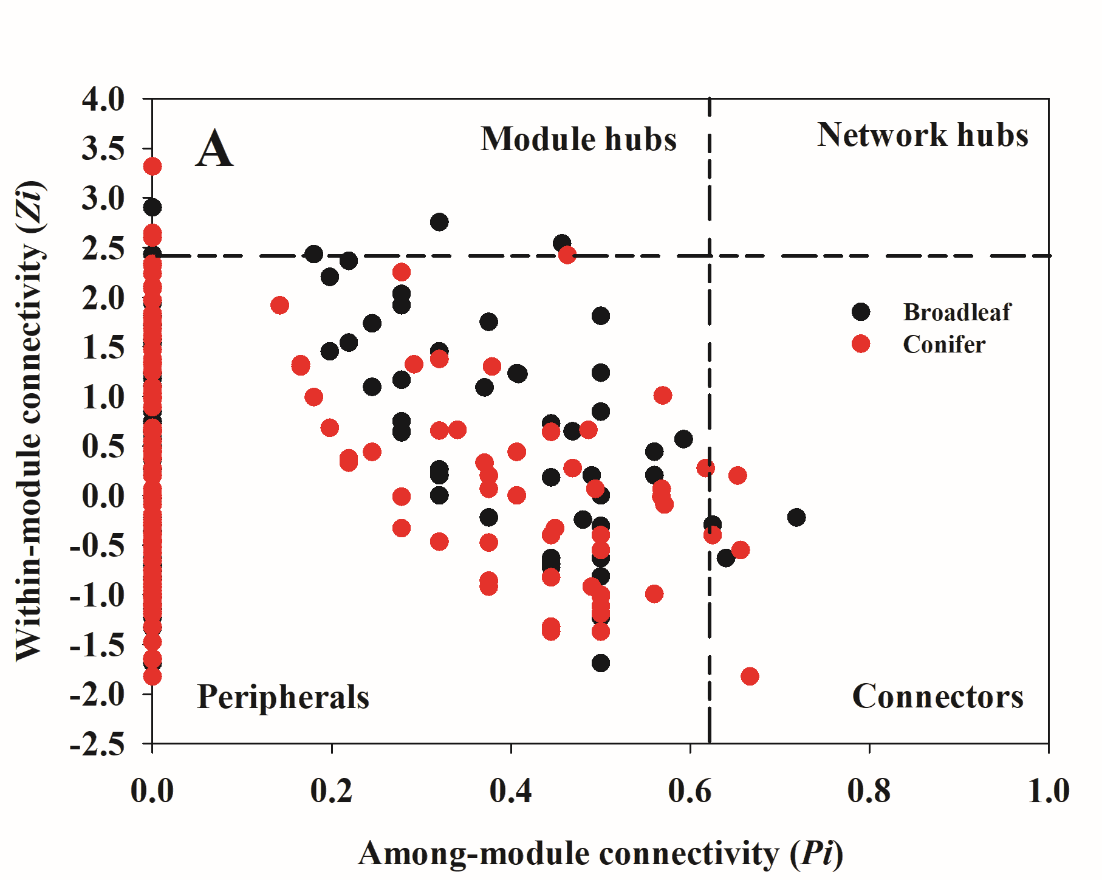


**Supplementary Fig. S4** Topological role of ASVs in the endophytic bacterial networks as displayed by the *Zi*-*Pi* plot. The nodes with *Zi* > 2.5 and *Pi* <0.62 are identified as module hubs, and those with *Zi* < 2.5 and *Pi* > 0.62 are connectors. The network hubs are determined by *Zi* > 2.5 and *Pi* > 0.62, and the peripherals are characterized by *Zi* < 2.5 and *Pi* < 0.62 as introduced by Olsen et al., 2007).


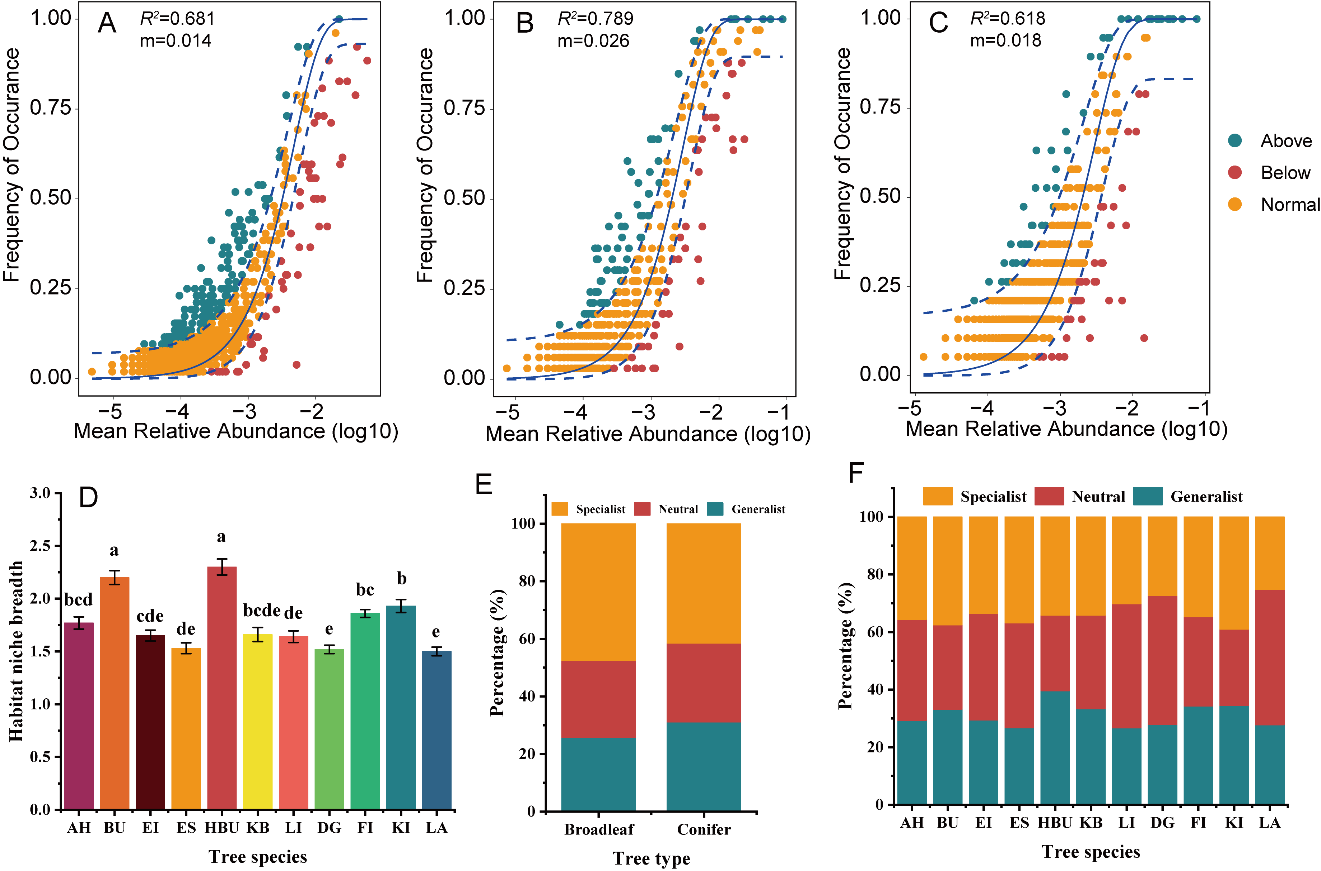


**Supplementary Fig. S5** Fit of the neutral community model (NCM) (A‑C) showing the ASVs predicted occurrence frequencies versus the relative abundance in endophytic bacteria. (A), all tree species; (B), broadleaf trees species; (C), coniferous tree species. ASVs that occur more frequently than predicted by the model are shown in blue, while those that occur less frequently than predicted are shown in red. ASVs that occur within the prediction are shown in yellow. The blue solid lines indicate the best fit to the Sloan’s neutral model and the blue dashed lines represent 95% confidence intervals around the model prediction. Comparison of mean habitat niche breadths (D) and relative contributions of habitat generalists and specialists (E, F) in all taxa in endophytic bacterial community among nine temperate tree species. Different letters indicate the significant difference among tree species (Turkey HSD test). *R^2^* and m values indicate the goodness of fit to the neutral model and the estimated migration rate, respectively.


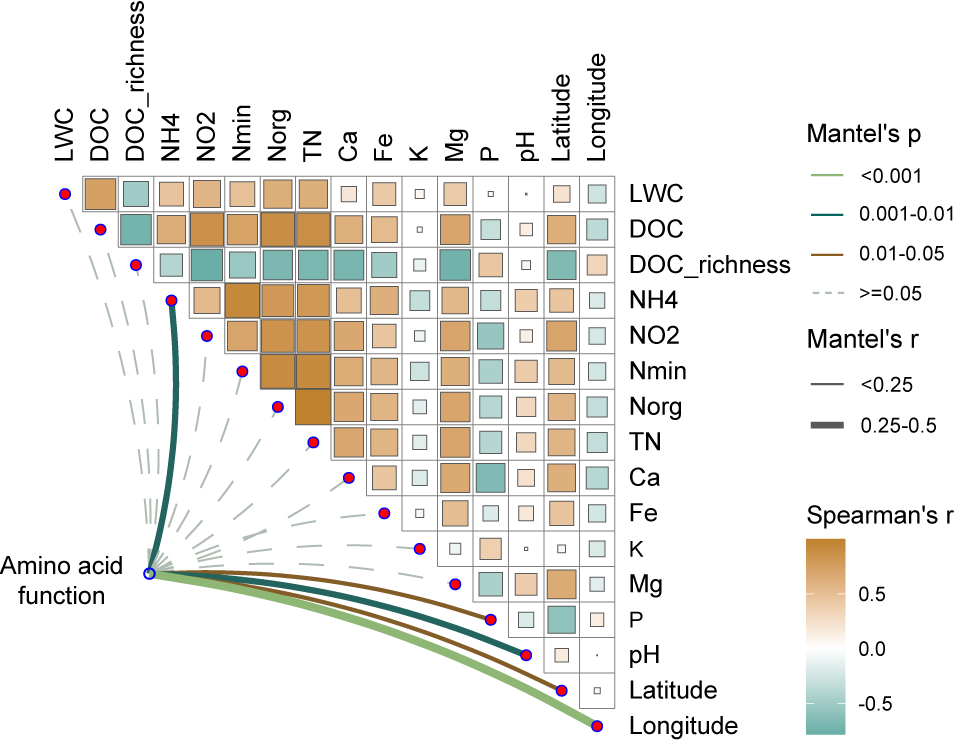


**Supplementary Fig. S6** Partial Mantel test to identify the main factors correlated to the amino acid function of bacteria. The leaf variables of endophytic bacterial community evaluated by partial Mantel tests based on the Bray–Curtis distance. Pairwise comparisons of leaf variables are shown in the upper-right area, which is represented with a color gradient using Spearman’s correlation coefficients. The edge width represents the partial Mantel’s r statistic for the corresponding correlation, and the edge color indicates that significance is tested following 999 permutations. LWC, leaf water content; DOC, dissolved organic carbon; NH_4_, ammonium nitrogen; N_min_, mineral nitrogen; N_org_, organic nitrogen; TN, total nitrogen.
